# Bioinformatics analysis reveals three key genes and four survival genes associated with youth-onset NSCLC

**DOI:** 10.1515/med-2022-0492

**Published:** 2022-07-06

**Authors:** Xuan Han, Peng Ren, Shaohua Ma

**Affiliations:** Department of Thoracic Surgery, Peking University Third Hospital, Haidian, Beijing 100191, China

**Keywords:** non-small cell lung cancer, youth-onset, bioinformatics, key genes, survival genes

## Abstract

Youth-onset non-small cell lung cancer (NSCLC) is a heterogeneous disease. It has a unique clinicopathology and special genetic background. In this study, three key genes, CDC20, CCNB2, and BUB1, have been identified in youth-onset NSCLC tumor tissues based on the TCGA and GEO cohorts. Functional enrichment analysis reveals that the “oocyte meiosis,” “cell cycle,” and the “P53 signaling pathway” are significantly enriched. Additionally, four survival genes, including AKAP12, CRIM1, FEN1, and SLC7A11, that affect the prognosis of youth-onset NSCLC patients are identified in this study. Finally, we construct a risk model to predict the overall survival of youth-onset NSCLC patients, the AUC of the risk model in 1, 3, and 5 years of overall survival is 0.808, 0.844, and 0.728. This study aims to provide a novel idea to explore the pathogenic genes of youth-onset NSCLC.

## 1 Introduction

Lung cancer is the second most common type of cancer worldwide, and it is also the most fatal malignancy [1]. GLOBOCAN forecasted 2.09 million new cases and 1.76 million deaths in 2018 [2]. Non-small cell lung cancer (NSCLC) is the most common subtype of lung cancer, accounting for over 85% [1,2]. With the development of targeted therapies and immunotherapy, adjuvant therapy may be beneficial for individuals with advanced NSCLC. Identifying the driver genes in the genetic background of NSCLC patients provides key diagnostic and therapeutic insight.

NSCLC is an age-related disease that is commonly diagnosed above the age of 65 [1,2]. However, young patients diagnosed with lung cancer have increased in recent years, and this group exhibits unique clinical characteristics [3,4]. In the current study, the relationship between youth-onset and genetic background has emerged as a new hot point in the field of NSCLC.

Youth-onset lung cancer is commonly defined as lung cancer that develops between the age of 18 and 50. It is characterized by a high female incidence, a hidden onset, and diagnosed at an advanced stage. Previous research has established that youth-onset patients have a higher rate of tumor gene mutation than their peers [5–8].

However, as a rare subtype of lung cancer, youth-onset NSCLC only accounts for approximately 2% of all patients [3,4,9]. Obtaining a sufficient number of tumor tissues for sequencing experiments at a single-center is challenging [10–12]. Fortunately, public databases, including The Cancer Genome Atlas (TCGA) and The Gene Expression Omnibus (GEO), provide sufficient sequencing information and clinical information of tumor tissues, which can help us to explore the genetic background of youth-onset NSCLC [13,14].

We downloaded the data from the public database and used it to perform a bioinformatics model to explore and validate the key genes and survival genes. The goal of our study is to identify key genes affecting tumor development and the patient’s survival. Our study also provides some novel strategies for the treatment and management of youth-onset NSCLC.

## 2 Materials and methods

### 2.1 Data collection and processing

We downloaded RNA-seq data of youth-onset NSCLC from the TCGA database (https://www.cancer.gov/tcga). The criteria include the following: (1) adult patients, (2) primary diagnosis of patients under the age of 50, and (3) tumor tissue must be pathologically diagnosed as NSCLC.

The GEO (https://www.ncbi.nlm.nih.gov/geo/) was extracted to screen studies on youth-onset NSCLC with the complete clinicopathology information. Our research includes GSE27262, GSE75037, and GSE102287. The R package “sva” was used to reduce the batch effect of three profiles from different platforms. All data sources are shown in Table 1.

**Table 1:** Sample enrolled in the study from TCGA and GEO databases

Database	Source	Sample		Plate form
		T	N	
TCGA	http://www.cancer.gov	61	5	
GSE27262	http://www.ncbi.nim.nih.gov/geo/query/acc.cgi?acc = GSE27262	7	7	Affymetrix Human Genome U133 Plus 2.0 Array
GSE75037	http://www.ncbi.nim.nih.gov/geo/query/acc.cgi?acc = GSE75037	5	5	Illumina Human WG-6 v3.0 expression beadchip
GSE102287	http://www.ncbi.nim.nih.gov/geo/query/acc.cgi?acc = GSE102287	5	5	Affymetrix Human Genome U133 Plus 2.0 Array

T: tumor tissues; N: para-carcinoma tissues.

To select differentially expressed genes by using the R package “limma,” the adjusted *p* < 0.05 and the |log2 (fold change)| ≥ 2.0 were regarded as cutoff values to identify the differential expression genes. Heatmap and volcano plots were used to illustrate the result.

### 2.2 Constructing protein–protein interaction (PPI) networks and identifying hub genes

STRING database (https://string-db.org/) was used to construct a PPI network. The PPI network was simplified by establishing the confidence interval (minimum required interaction score = 0.9) and hidden isolated proteins.

Cytoscape program (version 3.6.1) provides a visual interface for simplifying the PPI networks. CytoHubba provides five methods for estimating the key genes, including Edge percolated component (EPC), Maximal clique centrality (MCC), Maximal neighborhood component (MNC), Node connect degree (Degree), and Node connect closeness (Closeness). The Top10 was assessed by five means based on their importance in the PPI network.

### 2.3 Construction WGCNA analysis for youth-onset NSCLC

To assess gene–gene correlations, the R package “WGCNA” was used to create a WGCNA network. TCGA and GEO data were normalized by the rpkm method. We clustered TCGA and GEO data by using hierarchical clustering. Using a soft threshold for calculating the connection strengths between two transcripts, a topological matrix is generated to categorize genes in RNA-seq data. To ensure module credibility, we specified settings (deepSplit = 2, minModuleSize = 50, mergeCutHeight = 0.25) and merged the low-credibility gene modules using the dynamic tree cut approach. The Pearson correlation coefficient between the gene modules and the phenotype was calculated after getting the gene modules.

A gene with higher Module Membership (MM) and Gene Significance (GS) score in modules was considered to robust connection with phenotype. In our study, GS > 0.2, MM > 0.5, and *p* < 0.05 were performed as the criteria for the key genes. Finally, we insert the genes in the significance modules and the differential expression genes. The results were visualized by the Venn plot.

### 2.4 Analysis of enriched functions and pathways

DAVID database (https://david.ncifcrf.gov/) was used to perform GO and KEGG enrichment analyses. GO and KEGG entries with a *p* < 0.05 were determined to be differential gene enrichment entries, and all enrichment entries were shown using bar graphs.

### 2.5 Annotation of key genes

HPA database (https://www.proteinatlas.org) provides functional annotations for key genes, such as subcellular localization, protein function, molecular function, and biological processes.

### 2.6 GSEA analysis of key genes

We separated the TCGA RNA-seq data into two groups based on the median expression of important genes. We performed the GSEA analysis by using GSEA v3.0 (http://www.broad.mit.edu/gsea/). *p* < 0.05 and FDR < 0.25 are considered as the significance thresholds.

### 2.7 Analysis of survival

Univariate Cox regression analysis was performed to identify genes associated with the overall survival of youth-onset NSCLC patients (*p* < 0.05). Besides, multivariate Cox regression analysis was used to determine survival genes and construct a prognostic model. Additionally, we employed the time-dependent receiver operating characteristic (ROC) curve to evaluate the risk model’s prediction capacity at 1, 3, and 5 years of overall survival of youth-onset NSCLC patients. The following R packages were used in the survival analysis procedure: “survival,” “glmnet,” “survminer,” and “survivalROC.”

### 2.8 Statistical analysis

The statistical data were analyzed by the Kruskal–Wallis (K–W) test, Mann–Whitney *U* test, Chi-square test, and Fisher’s exact test. The univariate and multivariate Cox proportional hazard regression models were employed to assess the hazard ratio of the signature and clinical features. The Kaplan–Meier curve analysis along with log-rank test was conducted to evaluate the clinical outcomes of youth-onset NSCLC patients. The statistically significant difference was determined by “*p* < 0.05.”

## 3 Result

### 3.1 Identification of genes expressed differentially

**Figure 1:**
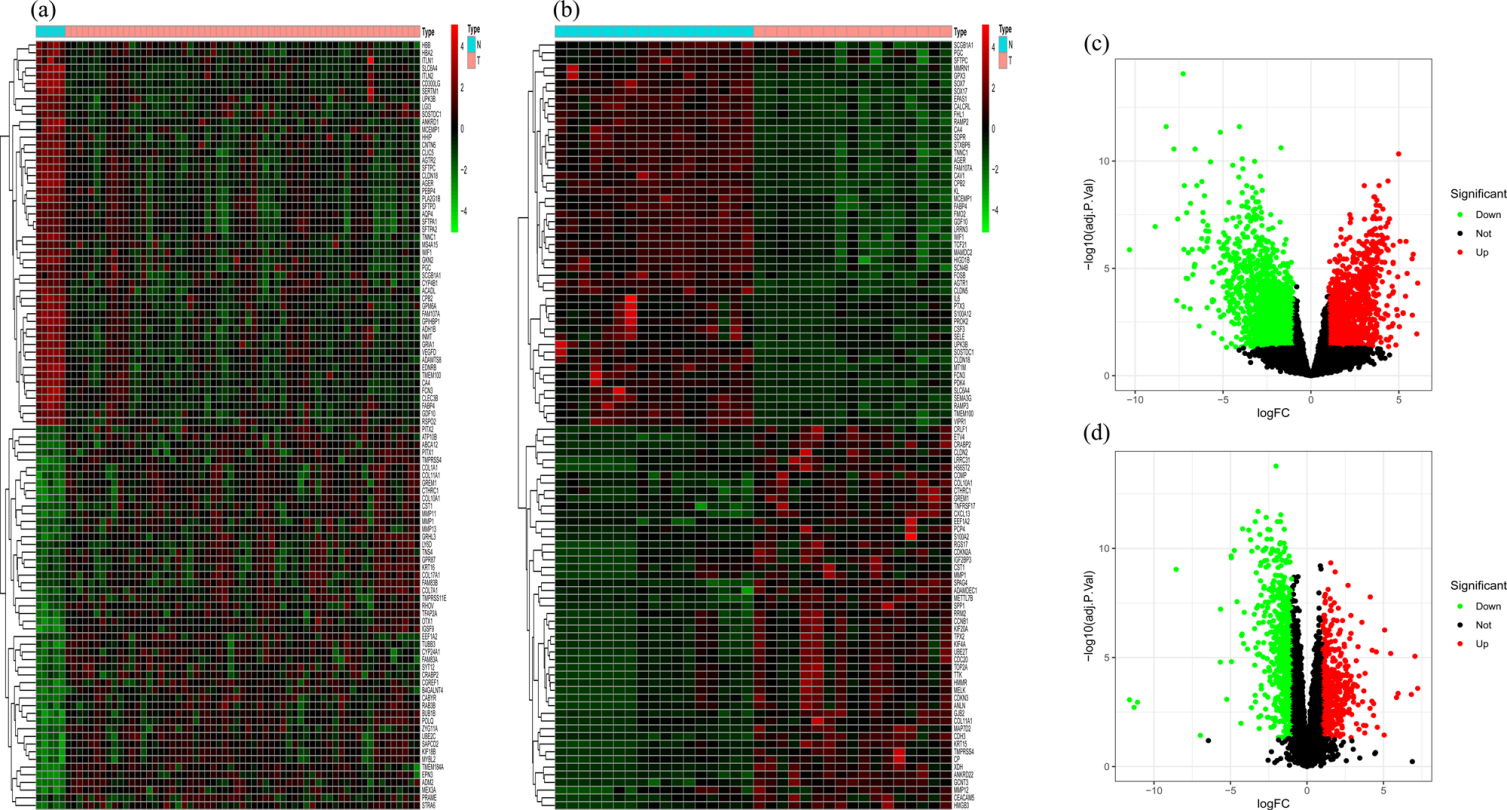
TOP50 differential expression genes in TCGA and GEO databases.

The TCGA array identified 1,147 genes that were upregulated and 2,002 genes were downregulated. In the GEO data, we identified 482 upregulated genes and 629 downregulated genes. The TOP50 differentially expressed genes in the TCGA and GEO databases were represented using heatmaps (Figure 1a and b) and volcano plots (Figure 1c and d).

### 3.2 Construction and analysis of PPI networks

**Figure 2:**
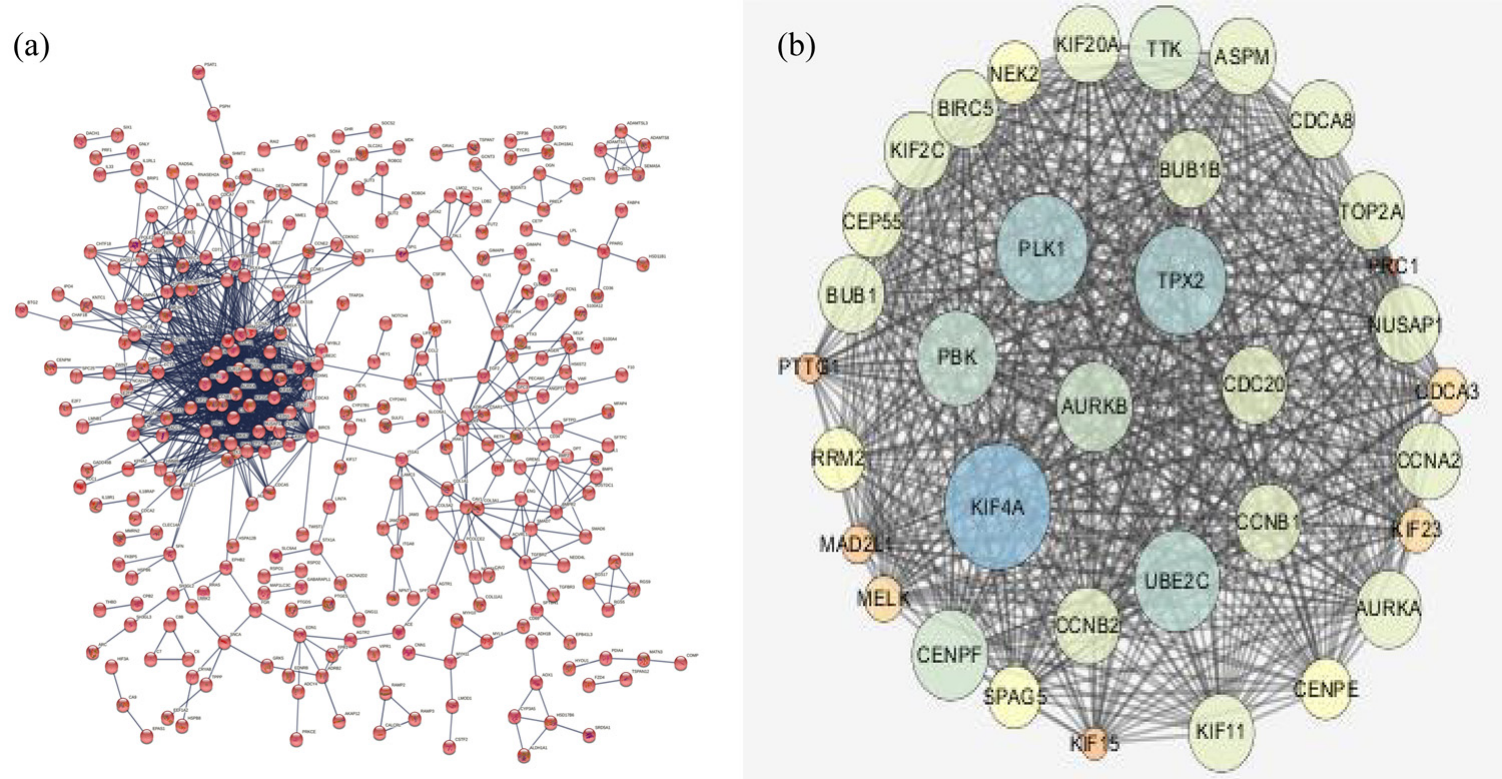
(a) PPI network involve 369 nodes and 1617 edges analysis by Cytoscape software. (b) Module1 included 35 nodes and 543 edges was regard as the key genes in the PPI network (MCODE score = 23.79).

The differential expression genes were constructed using a protein-interaction network with 369 nodes and 1,617 edges for key genes analysis (Figure 2a). Module 1 was determined to be the most significant module in the PPI network (MCODE score = 23.79). It included 35 nodes and 543 edges (Figure 2b). The degree size of Module 1 represents the importance of the protein linkage in the PPI network. Key genes were defined as the TOP10 genes in all five measures and are displayed in Table 2. As a result, we identified the key genes including CDC20, CCNB2, and BUB1.

**Table 2:** The TOP10 genes estimated by CytoHubba

**Category**	**EPC**	**MCC**	**MNC**	**Degree**	**Closeness**
1	CDKN3	***CDC20***	***CDC20***	***CDC20***	CCNA2
2	KIF2C	***CCNB2***	CCNA2	CCNA2	***CDC20***
3	AURKB	***BUB1***	***CCNB2***	***CCNB2***	***CCNB2***
4	***BUB1***	KIF2C	CCNB1	CCNB1	KIF11
5	MAD2L1	BUB1B	KIF11	KIF11	AURKB
6	***CDC20***	CCNB1	***BUB1***	***BUB1***	CCNB1
7	UBE2C	TOP2A	CDCA8	AURKB	TOP2A
8	***CCNB2***	KIF20A	AURKB	CDCA8	UBE2C
9	CENPE	CCNA2	TOP2A	TOP2A	***BUB1***
10	CDT1	NUSAP1	BUB1B	BUB1B	PLK1

Bold genes regarded as key genes in our study.

### 3.3 WGCNA analysis of youth-onset NSCLC

**Figure 3:**
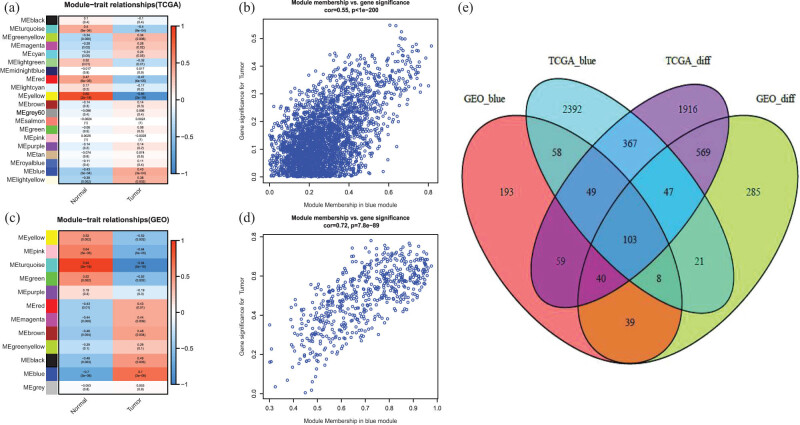
TCGA module-trait analysis (a) and module genes screen (b). GEO module-trait analysis (c) and module genes screen (d). (3) the key differential expression gene set (e).

We performed the WGCNA network, the module-trait analysis revealed that the blue module, which contains 3,045 genes, is the most significant module related to youth-onset NSCLC in TCGA array (Figure 3a). Additionally, genes having a higher GS and MM score in the blue module were identified as significant genes related to cancers (Figure 3b). In the GEO matrix, following module-trait analysis, the ME turquoise, blue, and pink modules were identified as critical modules (Figure 3c). The blue module, which contains 549 genes, was found to be the most strongly related to youth-onset NSCLC tumors (Figure 3d).

We got the intersection of the differently expressed genes and the significant genes in WGCNA analysis (Figure 3e). Naturally, we identified 103 genes including CDC20, CCNB2, and BUB1 under this category. These genes were further used in the function enrichment analysis.

### 3.4 Function enrichment analysis of the key genes

**Figure 4:**
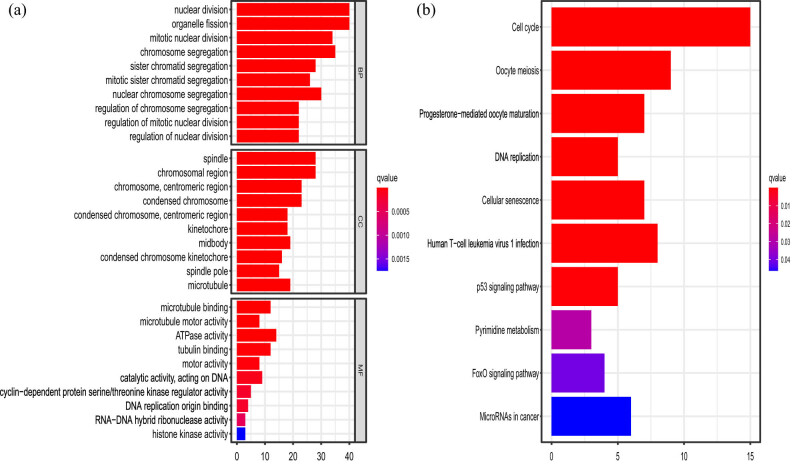
GO (a) and KEGG (b) pathways enriched in key genes.

GO analysis indicated that the key genes were highly concentrated in biological processes including nuclear division, organelle fission, mitotic nuclear division, and chromosomal segregation. As evidenced by cellular components, genes were plentiful in the spindle, chromosomal area, and chromosomal centromeric region of the chromosome. The molecular function was enriched in microtubule-binding, ATPase activity, and tubulin binding (Figure 4a).

KEGG analysis revealed the genes were enriched in signaling pathways including cell cycle, oocyte meiosis, and human T cell leukemia virus 1 infection. It is worth noting that the key genes were enriched in the biological signaling pathway P53, which is intricately associated with the youth-onset NSCLC (Figure 4b).

### 3.5 GSEA analysis for key genes

**Figure 5:**
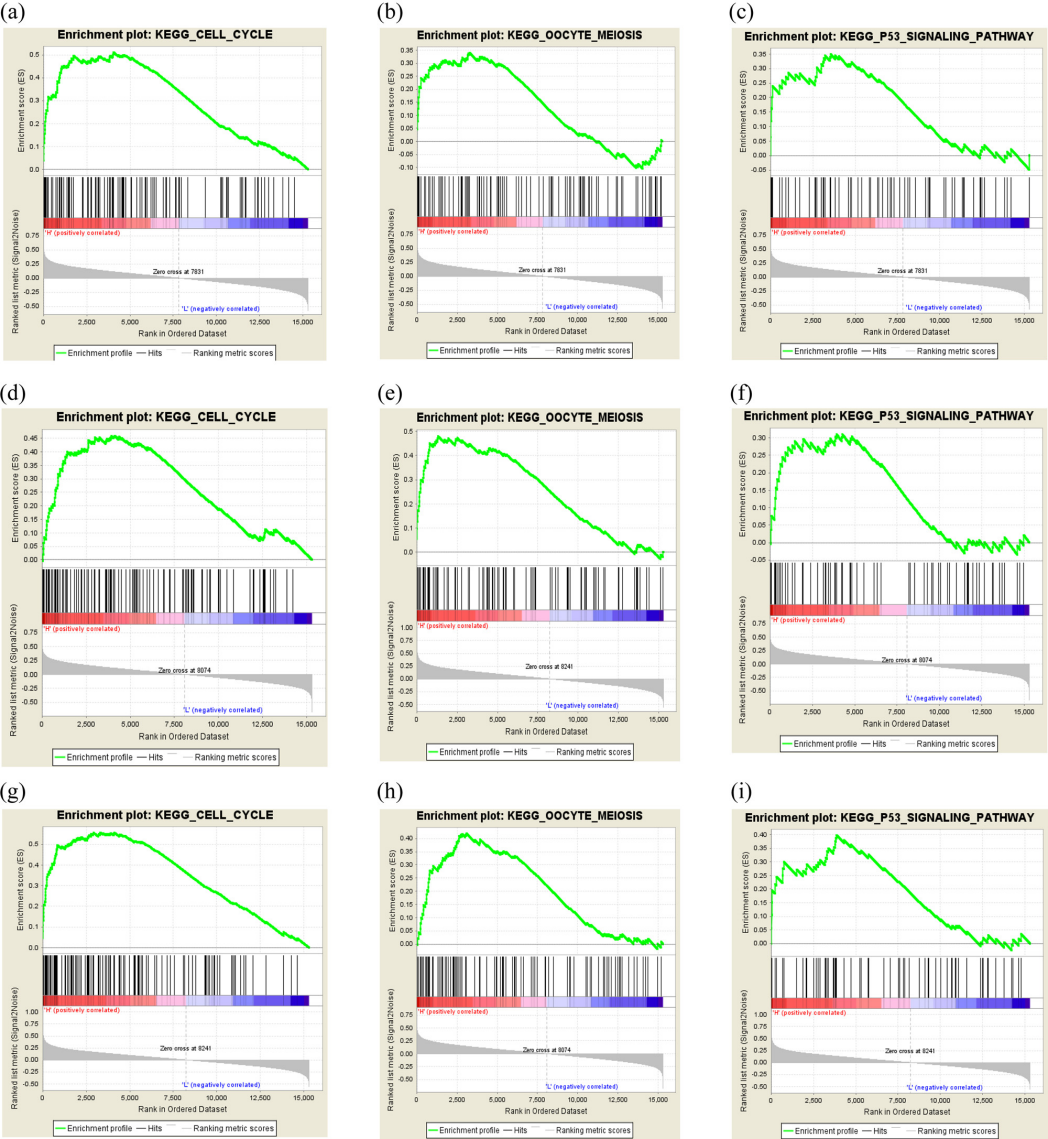
GSEA analysis of CDC20 (a–c), CCNB2 (d–f) and BUB1 (g–i).

We focused on the phenotypic regulation of key genes after aligning the TCGA data. The high-expression phenotypes of CDC20 (Figure 5a–c), CCNB2 (Figure 5d–f), and BUB1 (Figure 5g–i) were found to be enriched in the “cell cycle,” “oocyte meiosis,” and “P53 signaling pathway.” All of the entries have been linked to NSCLC.

### 3.6 Survival analysis

**Figure 6:**
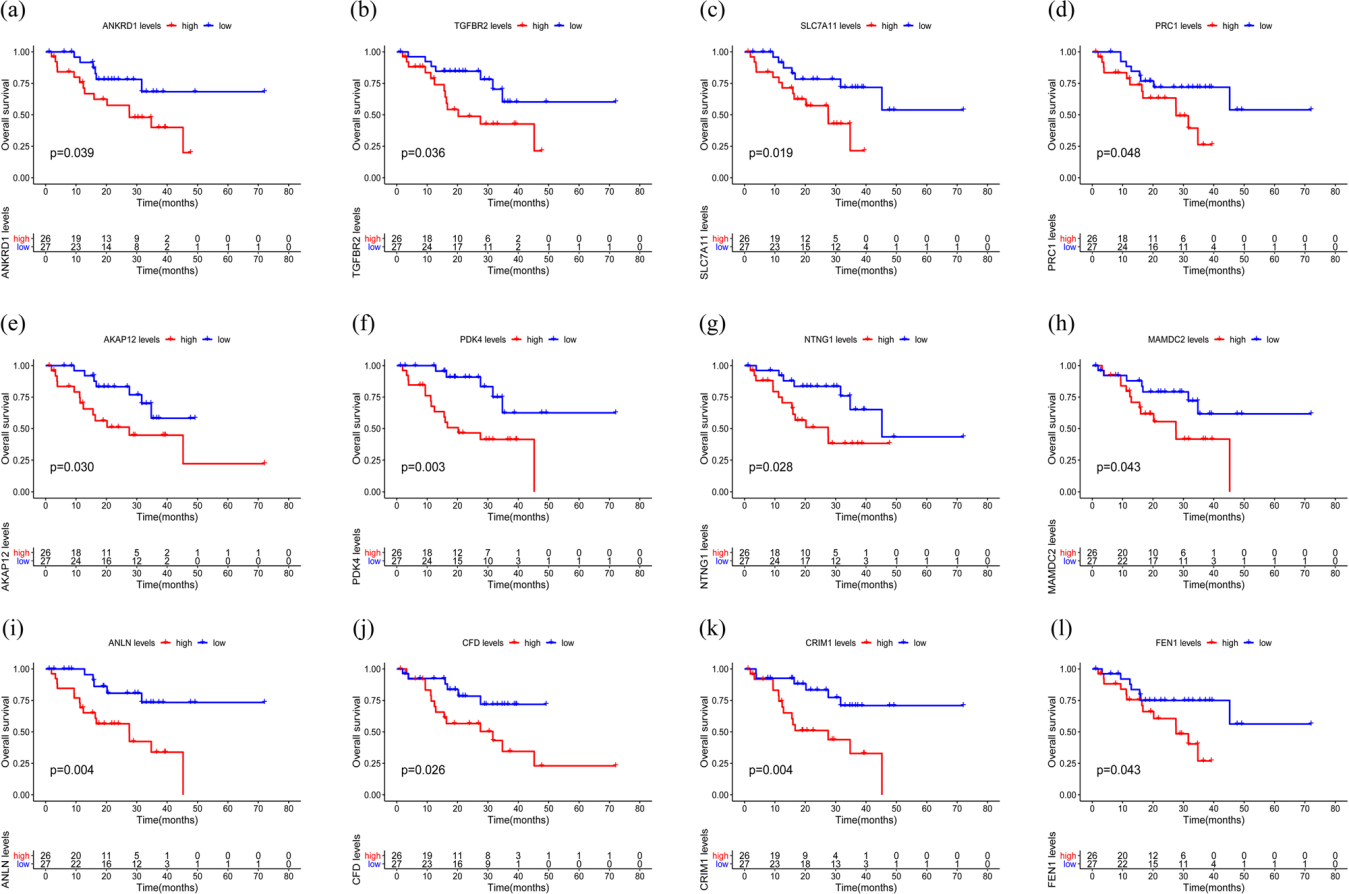
The Kaplan-Meier curve of 12 up-regulation genes in youth-onset NSCLC affect overall survival.

**Figure 7:**
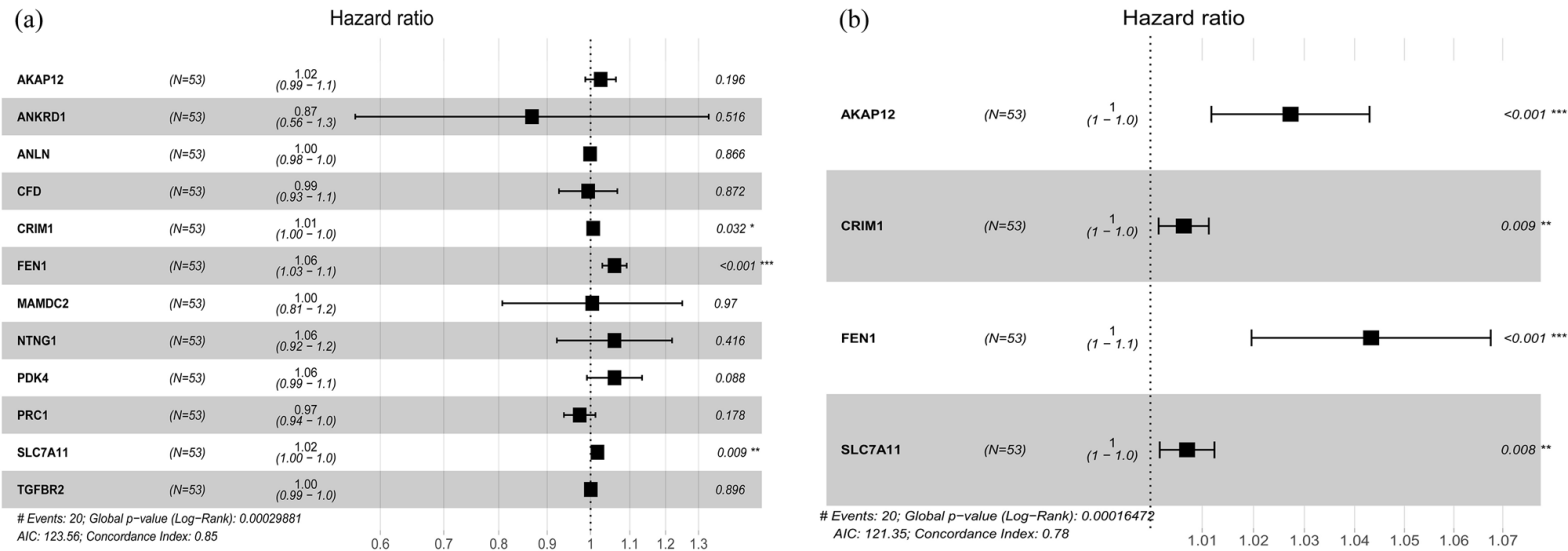
The risk prognostic model, included AKAP12, CRIM1, FEN1 and SLC7A11.

**Figure 8:**
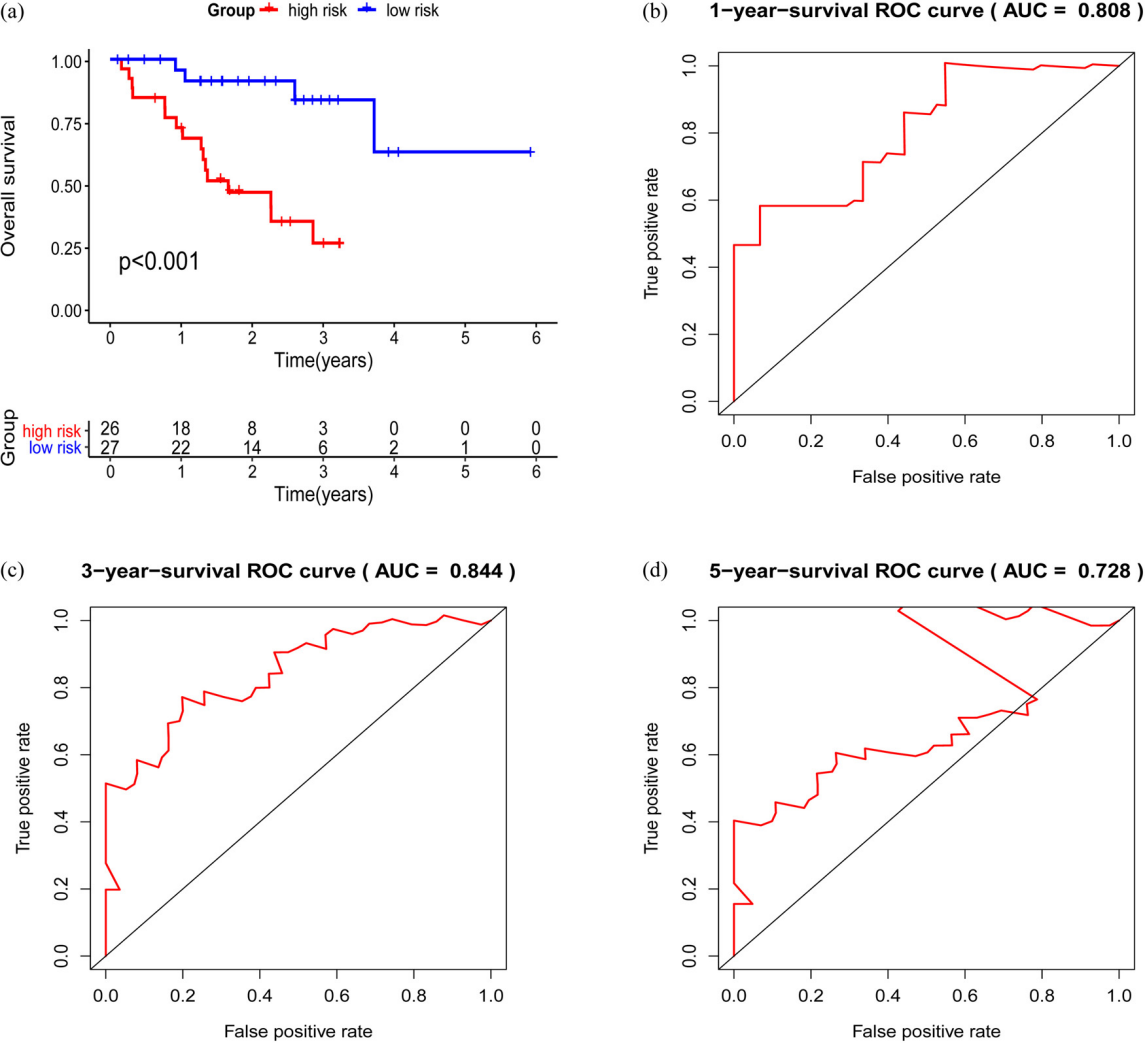
The ROC curve assess the predictive ability of the risk model at different endpoints (1, 3, and 5, years).

We discovered 12 upregulated genes in tumor tissues associated with poor prognosis (*p* < 0.05). The Kaplan–Meier curve is shown in Figure 6. We simplified the model by including the 12 survival genes and constructing the multivariate survival model. Finally, a total of four survival genes, including AKAP12, CRIM1, FEN1, and SLC7A11, were found for inclusion in the risk prognostic model (Figure 7). Kaplan–Meier curves revealed that the low-risk group was significantly better than the high-risk group in overall survival. The AUC of the ROC curves were 0.808, 0.844, and 0.728, which indicated the good prediction ability of the youth-onset NSCLC patients (Figure 8).

## 4 Discussion

Youth-onset NSCLC has a specific driver gene background, which is widely recognized in nowadays research [8,15]. However, the majority of research on the driver genes of youth-onset lung cancer is undertaken at single-center or small-panel NGS. Additionally, it is commonly accepted that ALK-positive plays an important role in the development and progression of youth-onset NSCLC [5,7,16]. Tian et al. revealed that, compared to senior NSCLC patients, youth-onset NSCLC patients are more likely to develop ALK fusion mutations and there are more young patients with unusual ALK fusion mutations. Youth-onset patients receiving crizotinib as first-line therapy have a better prognosis than their older competitors [17]. However, the positive rate of ALK mutations is about 5% in Asian NSCLC patients. While the ALK mutation is more prevalent in youth-onset, it still accounts for less than 10% of the NSCLC [18,19]. Our study aims to discover novel key genes that serve as molecular targets for youth-onset NSCLC and provide strategies for treatment.

In this study, we identified differential expression genes between tumor and paracancerous tissues in youth-onset NSCLC. Function enrichment analyses revealed that the differential expression gene was closely connected with cell cycle progression, which indicated that the abnormal cell cycle induced the development of youth-onset NSCLC. KEGG enrichment analysis also revealed that the classical TP53 signaling pathway had an important role to support the tumorigenesis.

WCGNA analysis focused on the interaction between the phenotypic characteristics of tumor and genes, narrowing down the possible candidate genes from another perspective. We finally identified that CDC20, CCNB2, and BUB1 were the key genes in the genetic background of youth-onset NSCLC.

CDC20 encoded the cell division circle 20 (CDC20) protein, which served as a regulatory protein throughout the cell cycle [20]. CDC20’s principal role is to activate the anaphase-promoting complex/cyclosome (APC/C) via the APC/C E3 ubiquitin ligase [21,22]. Nowadays, CDC20 is widely believed to be associated with a poor lung cancer prognosis [23,24]. Patients with lung cancer who test positive for CDC20 had a shorter OS and RFS, particularly those with NSCLC [25]. Shang et al. revealed that CDC20 deficiency is associated with suppression of cell proliferation, induction of apoptosis, and cell cycle arrest. The cellular function implies that CDC20 may be a potential target for tumor-targeted therapy development [26]. However, no research has assessed if increased CDC20 expression correlates with the age at NSCLC acquired. It has been demonstrated that CDC20 specificity in youth-onset NSCLC diagnosed and requires further studies.

Cyclin B2 (CCNB2), as a member of the cyclin family, plays a role in cell cycle regulation [27]. Unlike CCNB1, CCNB2 is a Golgi apparatus protein that binds to transforming growth factor β RII. It plays a vital function in cell cycle control mediated by TGF-β. According to recent studies, increased CCNB2 expression is related to a poor prognosis in patients with NSCLC [28–30]. Besides, high CCNB2 expression is associated with a poor prognosis in other organ tumors such as breast and bladder cancer. It also proves that decreasing CCNB2 expression can reduce the tumor’s invasion capacity and prevent distant metastasis [31].

BUB1 is a serine/threonine protein kinase that initiates spindle activity by phosphorylating the mitotic checkpoint complex. The main protein function is involved in cell mitosis and regulates the cell cycle [32]. Besides, previous studies revealed that BUB1 may be involved in the DNA damage response [33]. Overexpression of BUB1 in tumor tissues is also considered to be a biomarker for poor prognosis in tumor patients [34,35]. Han et al.’s research found that silencing BUB1 dramatically reduces the potential of cancer stem cells in the MDA-MB-231 breast cancer cell line. It means BUB1-targeted cancer stem cell therapy may represent potential treatment strategies [36].

We recruit 53 youth-onset NSCLC patients for screening survival genes and constructing risk models. We discovered four genes that are influencing survival in youth-onset NSCLC patients, including AKAP12, CRIM1, FEN1, and SLC7A11. Mathematical modeling can be used to assess the study’s effectiveness [37–39]. We construct a risk model to predict the survival of youth-onset NSCLC patients. Multivariable Cox regression analysis found that patients classified as high-risk had a statistically lower 1, 3, and 5 years of overall survival than their competitors.

This study has limitations, while we found tumor driver genes related to youth-onset lung cancer development and prognosis, the number of cases in the TCGA database remains insufficient. Due to the database’s continuous updating, we expect that additional samples will be accessible in the future to refine the studies.

## 5 Conclusion

In a summary, three key genes, including CDC20, CCNB2, and BUB1, are identified that closely connect with pathogenic mechanism of youth-onset NSCLC. We also explore the tumorigenesis of youth-onset NSCLC from the perspective of the biological signal pathway and phenotypic characteristics. We also constructed a risk model including four survival genes confirming good prediction ability in youth-onset NSCLC patients. Despite our study limitations by insufficient data, we provide a novel idea for future study on similar studies.

## Abbreviations

NSCLC: non-small cell lung cancer
TCGA: the cancer genome atlas program
GEO: gene expression omnibus
AUC: area under curve
PPI: protein-protein interaction
WGCNA: weighted correlation network analysis
GO: gene ontology
KEGG: kyoto encyclopedia of genes and genomes
GSEA: gene set enrichment analysis
FDR: false discovery rate
NGS: next-generation sequencing
OS: overall survival
RFS: relapse-free survival
ALK: anaplastic lymphoma kinase
